# Temperature-Dependent Affinity Changes in Substrate Binding Affect the Cleavage Activity of BthC2c1

**DOI:** 10.2174/0929866530666230125100320

**Published:** 2023-04-23

**Authors:** Dan Wu, Jieting Liu, Yong Liu, Yufei Qiu, Zhiqin Cao, Yu Pan, Jiayi Shi, Xiaohuan Yuan

**Affiliations:** 1 Heilongjiang Province Key Laboratory of Anti-fibrosis Biotherapy, Mudanjiang Medical University, Mudanjiang, 157011, Heilongjiang, China;; 2 College of Life Sciences, Mudanjiang Medical University, Mudanjiang, 157011, Heilongjiang, China;; 3 Center for Comparative Medicine, Mudanjiang Medical University, Mudanjiang, 157011, Heilongjiang, China

**Keywords:** CRISPR-Cas, C2c1, cleavage activity, Cpf1, *E. coli* C43, BthC2c1

## Abstract

**Background:**

The CRISPR-Cas system is an adaptive immune mechanism for bacteria and archaea to resist foreign invasion. Currently, Cas9 and Cpf1 have been widely studied and applied in gene editing. C2c1 is a newly discovered CRISPR-Cas system endonuclease. It has broad application prospects due to its small molecular weight and high substrate recognition specificity.

**Objectives:**

*Bacillus thermoamylovorans* C2c1(BthC2c1) was expressed in *E. coli* C43 (DE3) competent cells, purified, and the BthC2c1-sgRNA-dsDNA complex was assembled. The effect of temperature on the cleavage ability of the BthC2c1 system was investigated.

**Methods:**

The cDNA of BthC2c1 was cloned into the vector pGEX-6P-1. BthC2c1 was expressed in *E. coli* C43(DE3) cells and purified using a GST affinity column and FPLC. The sgRNAs were transcribed and purified *in vitro*, and the complexes were assembled by gel filtration chromatography. The enzyme cleavage activity of BthC2c1 at different temperatures was investigated using an *in vitro* cleavage assay. Microscale Thermophoresis detected the affinity of the BthC2c1-sgRNA complexes to substrate DNA.

**Results:**

BthC2c1 proteins were prokaryotically expressed and purified. The complex of BthC2c1 with sgRNA and dsDNA was assembled. *In vitro* cleavage assay results showed that BthC2c1 cleaved the target DNA at temperatures ranging from 37°C to 67°C. The cleavage ability of BthC2c1 at 42^°^C was stronger than that at 37^°^C. The results of affinity detection showed that the affinity between the BthC2c1-sgRNA complex and ds36/36 at 42^°^C was stronger than that at 37^°^C.

**Conclusion:**

In this study, BthC2c1 was expressed, purified, and assembled into a complex with sgRNA and dsDNA. BthC2c1 cleaved DNA within the temperature range of 37^°^C to 67^°^C. The affinity of BthC2c1-sgRNA to DNA at 42°C was significantly enhanced than that at 37°C. It may be related to its stringent substrate recognition pattern, which differs from Cas9 and Cpf1. The temperature-dependent affinity changes of substrate binding may be part of the reason for the stronger cleavage activity of BthC2c1 at 42^°^C. This study may provide an experimental basis for optimizing and modifying the C2c1 gene editing system.

## 1 INTRODUCTION

Clustered, regularly interspaced short palindromic repeats (CRISPR)/Cas systems are a defense system for archaea and bacteria that can be used to resist bacteriophage invasion [1, 2]. The CRISPR/Cas system consists of two key components: one is the adaptation module, which sends the genetic material into the CRISPR array to make CRISPR RNA (crRNA), and the other is the effector module, which is guided by the crRNA to target and cleave invading nucleic acids. According to the class of effect module structure, the CRISPR-Cas system can be divided into two classes [3-5]. The first class type of the CRISPR/Cas system includes type I, type III, and the putative type IV, which form multi-subunit effect complexes characterized by the combination of crRNA with a wide range of Cas proteins. The second class type of the CRISPR/Cas system includes type II, V, and VI systems, which are characterized by the binding of crRNA to a single Cas protein. The most representative effector protein of the class 2 type II CRISPR/Cas system is CRISPR/Cas9. Cas9 is a large multi-region nuclease that conservatively recognizes and cleaves the target DNA by recognizing the adjacent protospacer motif (PAM) sequence on the target DNA [6-10]. Cas9 is the most widely studied CRISPR/Cas system and has been designed as a gene editing tool based on its RNA-guided endonuclease activity [11-13]. C2c1 belongs to the v-B subtype and is called Cas12b [14]. C2c1 is a DNA endonuclease guided by crRNA and trans-activating crRNA (tracrRNA) with double-stranded DNA cleavage activity [15]. C2c1 mediates DNA interference by recognizing T-rich PAM at the end of the original interval sequence [16].

To reveal the molecular mechanism of single-guide RNA (sgRNA) -mediated C2c1 recognition of DNA and cleavage of target DNA, the author previously studied the structure and function of the ternary complex of *Bacillus thermoamylovorans* C2c1(BthC2c1) with sgRNA and dsDNA. The study revealed the mode of BthC2c1 recognition of dsDNA, particularly the stringent recognition mode of PAM DNA by BthC2c1. This pattern differs from the relatively loose PAM recognition pattern of other CRISPR-Cas nucleases like *Staphylococcus aureus* Cas9 (SaCas9) and Cpf1. Compared with SaCas9 and Cpf1, BthC2c1 may recognize substrates more specifically. Two separate research groups analyzed the structure of the ternary complex of *Alicyclobacillus acidoterrestris* C2c1 (AacC2c1) with sgRNA and dsDNA and the structure of the binary complex of AacC2c1 and sgRNA. The AacC2c1-sgRNA system is highly sensitive to single nucleotide mismatches between the RNA and target DNA, suggesting that AacC2c1 may have the advantage of low off-target risk. Moreover, the assembly mechanism of AacC2c1 sgRNA is different from Cas9 and Cpf1 [17,18]. Sequence alignment and complex structure comparison of BthC2c1 and AacC2c1 were performed. The results showed that BthC2c1 and AacC2c1 had 33% sequence identity [19]. The structural comparison revealed that the structure of BthC2c1 was conserved with that of AacC2c1, and the recognition patterns of sgRNA and dsDNA by BthC2c1 and AacC2c1 were very similar.

Since the optimal cleavage temperature of C2c1 is higher than 37°C, it is difficult to apply to gene editing in mammals. On the other hand, CRISPR/AaC2c1 was developed as a gene-editing tool for editing mammals [20]. In addition, a series of C2c1 homology systems have been designed for editing mammalian cell genomes. Efficient multiplex genome editing was performed using TcC2c1 and a cognate sgRNA [21]. Other studies have optimized BhC2c1 v4 and BvC2c1, which have been confirmed to be two novel proteins that can also be edited in human cell lines [22].

Based on the previous structural and functional research, here we show that BthC2c1 was expressed and purified, and the complex of BthC2c1 with sgRNA and dsDNA was assembled. The cleavage activity of CRISPR-BthC2c1 at different temperatures was detected by *in vitro* cleavage assay. The affinity of CRISPR-BthC2c1 to the substrate at different temperatures was investigated by Microscale Thermophoresis (MST). This study may provide an experimental basis for optimizing the BthC2c1 system for gene editing.

## 2 MATERIALS & METHODS

### 2.1 Plasmid Construction and Protein Expression

BthC2c1 cDNA was synthesized and cloned into a bacterial expression vector pGEX-6P-1 (GE Healthcare, Chicago, USA). The vector has a GST tag at the N-terminus and a PreScission Protease cleavage site between the GST tag and BthC2c1. BthC2c1 was expressed in *E. coli* C43 (DE3) competent cells (Novagen, Vadodara, Gujarat). Positive clones were picked and inoculated in 5 mL of LB medium. The recombinant protein was induced by isopropyl β-D-1-thiogalactopyranoside (IPTG). The optimal expression conditions were screened according to different induction temperatures, the OD value, and induction time. The cells were collected by centrifugation at 4000 rpm for 10 minutes. Bacteria were resuspended and sonicated. The supernatant, after centrifugation was detected for protein expression using 12% sodium dodecyl sulfate-polyacrylamide gel electrophoresis (SDS-PAGE).

### 2.2 Purification of Recombinant Protein

Positive clones were selected and placed into 5 mL of LB liquid medium with ampicillin resistance, cultured at 37°C for 12 hours, transferred to 1 L of LB liquid medium with ampicillin resistance, and cultured at 37°C to an OD600 of about 0.8 The temperature was lowered to 16°C and 0.3 mM isopropyl-β-D-1-thiogalactopyranoside (IPTG, BioFroxx, Germany) was added for induction overnight.

Since the BthC2c1 protein was tagged with GST, GST Sepharose (GE Healthcare) was used for purification. The bacterial solution was centrifuged and resuspended, and 2 mM of the protease inhibitor phenylmethylsulfonyl fluoride (PMSF, Solarbio, Beijing, China) was added. The bacterial solution was sonicated and centrifuged at 15,000 rpm at 4°C for 40 minutes. The supernatant was loaded onto a gravity column with 2 mL of GST Sepharose and washed with a salt (NaCl) gradient to remove non-specifically bound proteins. A total of 5 mL of washing buffer was added, the gravity column was blocked, and 80 μL of PreScission Protease was added to digest the proteins overnight at 4°C. The next day, 5 mL of washing buffer was added, and the flow-through protein was collected. The digested protein was purified by heparin affinity chromatography (Heparin, GE Healthcare). Heparin Buffer A is 25 mM Tris (pH 8.0) and 3 mM DTT. Heparin Buffer B is 25 mM Tris (pH 8.0), 1 M NaCl and 3 mM DTT. The protein at the peak tip was selected for further purification by ion exchange chromatography (Resource S, GE Healthcare). Resource S Buffer A is 25 mM Tris (pH 7.6) and 3 mM DTT. Resource S Buffer B is 25 mM Tris (pH7.6), 1 M NaCl and 3 mM DTT. Peak proteins were detected by 12% SDS-PAGE.

### 2.3 *In Vitro* Transcription and Purification of sgRNA

sgRNAs were transcribed *in vitro* using T7 RNA polymerase. The transcription system was as follows: 0.1 M HEPES-K pH 7.9; 12 mM MgCl_2_; 30 mM DTT; 2 mM Spermidine; 2 mM NTP; 80 μg/mL T7 RNA polymerase; and 500 nM template. All the other reagents were preheated to 37°C before adding the template and T7 polymerase. All the components were mixed and reacted at 37°C for 6 hours. The reactions were terminated by freezing at -80°C for 1 hour. Pyrophosphate was precipitated by Mg^2+^, and the DNA template was precipitated by spermidine. The precipitates were removed, and RNA was precipitated by ethanol. The RNA pellet was resuspended and purified by electrophoresis on a denaturing polyacrylamide gel (8 M urea). Further extraction was performed using the Elutrap electro-elution system. Ethanol precipitation was performed, the RNA was resuspended in diethyl pyrocarbonate treated water (DEPC.H_2_O), and RNA quality was detected using a urea polyacrylamide gel.

### 2.4 Ternary Complex Assembly

The two DNA single strands were BthC2c1-P-12 (GTGTGGATTCCG) and BthC2c1-29 (ATTAAATGACT-TCTCCCCGGAATCCACAC). The targeting and non-targeting DNA strands were mixed in an equimolar ratio, and the two single DNA strands were denatured at 95°C for 3 minutes and then slowly cooled to room temperature to form dsDNA. To assemble the BthC2c1-sgRNA-dsDNA ternary complex, BthC2c1 protein, sgRNA, and dsDNA were mixed and incubated at room temperature for 10 minutes, and then incubated at 4°C for 40 minutes to fully bind. The complexes were purified by gel filtration chromatography (HiLoad 16/600 Superdex200, GE Healthcare) to remove the excess sgRNA and dsDNA. Complexes were detected by 12% SDS-PAGE and a 10% denaturing TBE-Urea gel.

### 2.5 *In Vitro* Cleavage Assay

The optimal temperature range for DNA cleavage by BthC2c1 needs to be determined. The substrate used in the *in vitro* cleavage assay included 5'-ATTC-3' PAM and a 19bp Protospacer (ATTCCGGGGAGAAGTCATTTAAT) of the human *EMX1* gene, which was ligated into the PUC18 vector. The primers were designed upstream and down-stream of the target sequence for PCR amplification, and a fragment of about 240bp containing 5'-ATTC-3' PAM and Protospacer was obtained. The cleavage reaction system was as follows: 300 ng substrate, 2.7 μM sgRNA, 400 nM BthC2c1 protein, 1.5 μL NEB buffer 3.1, 5 mM DTT, and DEPC water for a total volume of 15 μL. Seven tempe-ratures, including 37°C, 42°C, 47°C, 52°C, 57°C, 62°C, and 67°C were utilized. The cleavage reaction was carried out at different temperatures for 30 minutes. After the reaction was terminated, 2× RNA loading buffer was added, and the samples were denatured at 95°C for 3 minutes. The cleavage activity of BthC2c1 was detected using 10% urea polyacrylamide gel electrophoresis.

### 2.6 Binary Complex Assembly

To assemble the BthC2c1-sgRNA complex, the BthC2c1 protein and sgRNA were mixed and incubated at room temperature for 10 min, and then incubated at 4°C for 40 min. The complexes were assembled by gel filtration chromatography (HiLoad 16/600 Superdex200, GE Healthcare). The peak protein was collected and detected by 12% SDS-PAGE and 10% denaturing TBE-Urea gel.

### 2.7 Affinity Detection Between BthC2c1mut-sgRNA and Substrate

The long-chain DNA used in the previous study was 29 bp. Sanger sequencing revealed that the BthC2c1 fragmented DNA product had a 7 nt 5’ overhang. This staggered double-strand break occured at the 16^th^ nucleotide of the non-target DNA strand and the 23^rd^ nucleotide of the target DNA strand away from the PAM. The break site on the target DNA strand was located beyond the guide RNA: target DNA heteroduplex fragments. The substrate DNA for affinity detection was extended on the previous DNA strand, including the break site. The sequences were: BthC2c1-P-36 (GTGTGGATTCCGGGGAGAAGTCATTTAATGAATTCG) and BthC2c1-36 (CGAATTCATTAAATGACTTC-TCCCCGGAATCCACAC). The two ssDNA were annealed at 95°C for 3 min and then slowly cooled to room temperature to form dsDNA36/36.

Microscale thermophoresis (MST) can detect molecule changes by measuring molecules' movement across microscopic temperature gradients. It can quickly analyze the interactions of biological samples, including proteins with proteins, proteins with small molecules, and small molecules with small molecules. The BthC2c1mut-sgRNA complex was labeled with a fluorescent marker (NT-647). The concentration of the NT-647-labeled complex at 100 nmol/L was kept unchanged, while the unlabeled dsDNA36/36 was serially diluted at an initial concentration of 2500 nmol/L. After mixing the reactive molecules, the sample was in equilibrium for 5 min. The capillary tubes were placed into the sample and loaded automatically by the capillary phenomenon and then placed on the capillary tray. The MST reaction temperature was set to 37°C and 42°C to detect the affinity of the BthC2c1mut-sgRNA complex to the substrate DNA.

## 3 RESULTS

### 3.1 Prokaryotic Expression and Purification of BthC2c1 Protein

**Figure 1:**
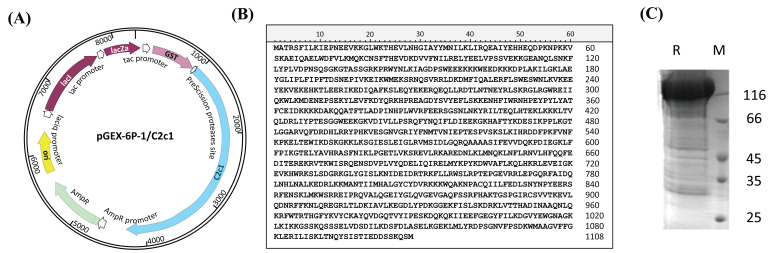
Vector construction and protein expression and preliminary purification of BthC2c1. (**A**) Map of pGEX-6P-1/C2c1 vector. (**B**) Sequence composition of BthC2c1. (**C**) SDS-PAGE electrophoresis results of BthC2c1 preliminary purified protein, R: BthC2c1 protein, M: marker.

**Figure 2:**
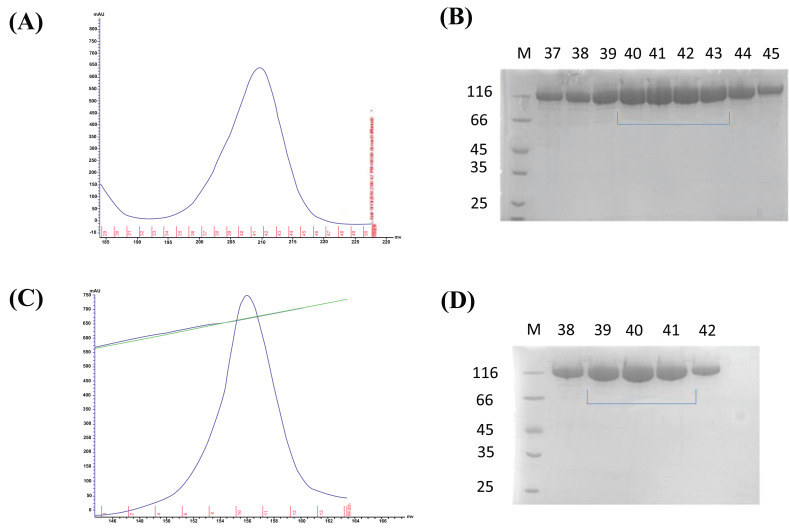
Further purification of BthC2c1 protein by FPLC. (**A**) Peak profile of BthC2c1 protein purified by Heparin, with UV absorption (mAU) on the vertical axis and elution volume (mL) on the horizontal axis. (**B**) SDS-PAGE electrophoresis results of BthC2c1 protein purified by Heparin. (**C**) Peak profile of BthC2c1 protein purified by Resource S. (**D**) SDS-PAGE electrophoresis results of BthC2c1 protein purified by Resource S.

The prokaryotic expression vector pGEX-6P-1/C2c1 was constructed (Figures **1A** and **1B**). The BthC2c1protein was highly expressed in *E. coli* by low-temperature induction (Figure **1C**). The BthC2c1 recombinant protein was initially purified with GST affinity column and digested with PreScission Protease overnight to remove the GST tag (Figure **1C**). After the overnight digestion, the BthC2c1 protein was further purified by FPLC on Heparin Column. Each fraction was 2 mL, and the peak samples were examined by 12% SDS-PAGE. Based on the observed peak and the result of the SDS-PAGE, the peak proteins with high purity, high concentration and good molecular behavior were selected for purification by ion exchange chromatography. 8 mL of fractions 40, 41, 42, and 43 were diluted to 40 mL with Resource S Buffer A (Figures **2A** and **2B**). The conductivity was about 6 mS/cm, and a cation exchange chromatography column (Resource S) was used for further purification. SDS-PAGE detected the peak fractions, and the fractions of 39, 40, and 41 were collected and used for the next experiment. (Figures **2C**and **2D**).

### 3.2 Assembly and Purification of BthC2c1-sgRNA-dsDNA

**Figure 3:**
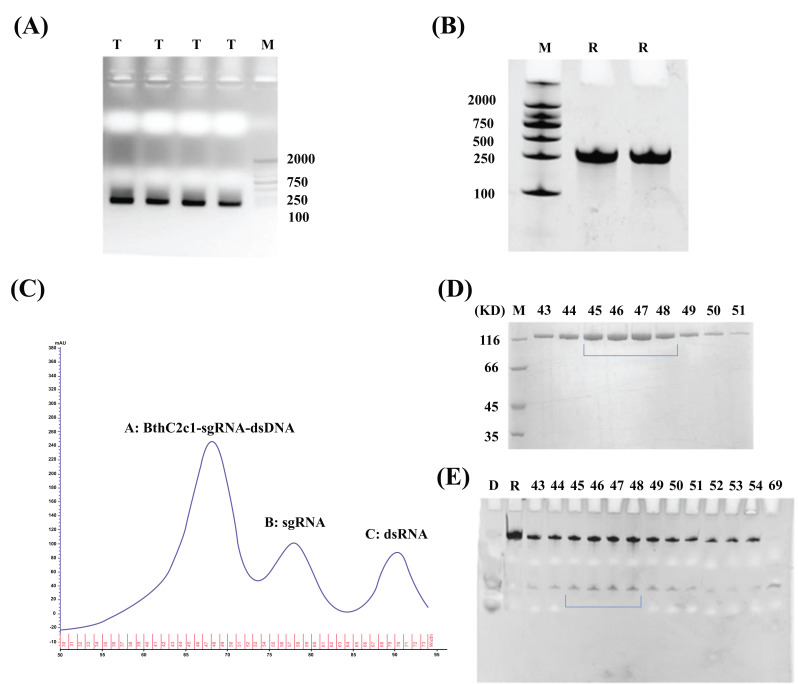
Assembly of the BthC2c1-sgRNA-dsDNA complex. (**A**) Template DNA for sgRNA transcription *in vitro*. (**B**) Denature gel electrophoresis results of BthC2c1 sgRNA. (**C**) Peak profile of the complex of BthC2c1-sgRNA-dsDNA assembled by gel filtration chromatography. (**D**) SDS-PAGE results of the complex purified by HiLoad. (**E**) Denature gel results of the complex purified by HiLoad.

The DNA template was obtained by PCR amplification. The BthC2c1 sgRNA was transcribed *in vitro* by T7 RNA polymerase and purified (Figures **3A** and **3B**). Assembly of BthC2c1 complexes with sgRNA and dsDNA was performed by gel filtration column. The results showed that the BthC2c1 protein had a strong interaction with sgRNA and dsDNA. The SDS-PAGE and denaturing gel electrophoresis results revealed that the protein and sgRNA appeared together with the dsDNA (peak A, in Figure **3C**), and the redundant RNA and DNA (peak B, peak C, in Figure **3C**) were removed (Figures **3C**-**3E**). The optimal ratio of protein and nucleic acid was determined by gel filtration chromatography. The optimal molar ratio of BthC2c1 protein to sgRNA and dsDNA is 1:1.4:1.5 Protein and nucleic acid are fully bound, and only a small amount of nucleic acid was excessive.

### 3.3 *In Vitro* Cleavage Activity of BthC2c1 in a Certain Temperature Range

**Figure 4:**
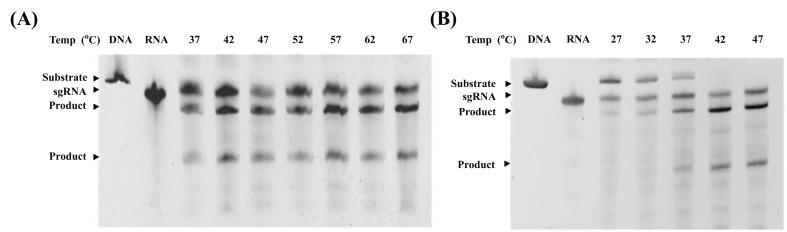
Detection of cleavage activity of BthC2c1 at different temperatures. (**A**) *In vitro* cleavage assay with a temperature gradient from 37 to 67°C. (**B**) *In vitro* cleavage assay with a temperature gradient from 27 to 47°C.

After adding the different groups of samples in parallel according to the cleavage reaction system, each group of samples was placed at different temperatures, including 37°C, 42°C, 47°C, 52°C, 57°C, 62°C, and 67°C. The cleavage activity of BthC2c1 under the different temperatures was detected using 10% urea polyacrylamide gel electrophoresis (Figure **4A**). The results showed that the substrates were cleaved in each group, and two clear-cut product bands were observed. Thus, BthC2c1 had DNA cleavage activity at a temperature range from 37^°^C to 67^°^C.

To test whether BthC2c1 was still active at lower temperatures. We set five temperature gradients of 27°C, 32°C, 37°C, 42°C, and 47°C. In order to compare the differences in cleavage activity at different temperatures, the reaction time was shortened to 15 min. The cleavage activity was detected by 10% urea polyacrylamide gel electro-phoresis (Figure **4B**). The cleavage activity of BthC2c1 was extremely weak at 27^°^C and 32^°^C, while it was slightly improved at 37^°^C, but the substrate DNA was not completely cleaved. The cleavage activity of BthC2c1 was significantly increased at 42°C and 47°C, and the substrate DNA was completely cleaved, revealing two clear enzyme cleavage product bands. The results showed that 42^°^C might be a suitable temperature for BthC2c1 cleavage activity.

### 3.4 Determination of the Affinity of the BthC2c1-sgRNA to the Substrate ds36/36

**Figure 5:**
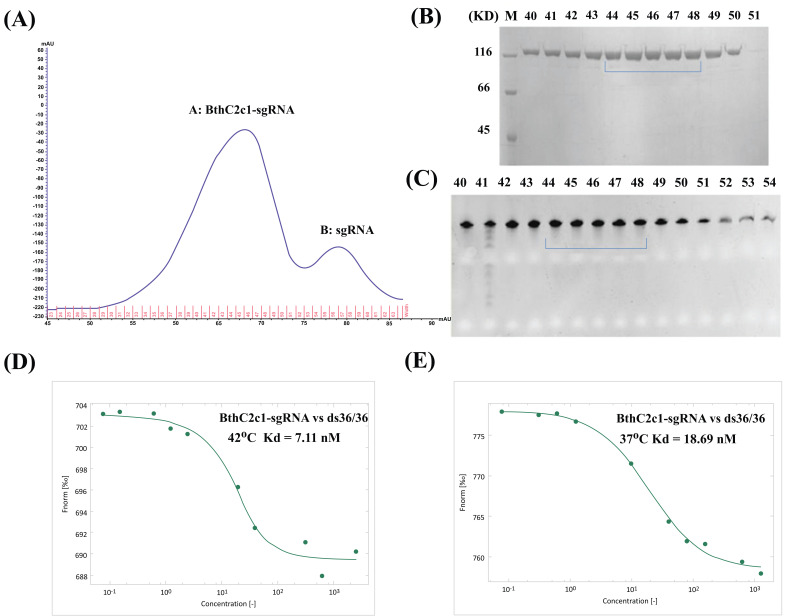
Affinity determination of BthC2c1-sgRNA complexes with substrates by MST. (**A**) Peak profile of the complex of BthC2c1-sgRNA assembled by gel filtration chromatography. (**B**) SDS-PAGE results of BthC2c1-sgRNA complex purified by HiLoad. (**C**) Denature gel results of BthC2c1-sgRNA complex purified by HiLoad. (**D**) The affinity of BthC2c1mut-sgRNA complex with ds36/36 at 42^°^C. (**E**) The affinity of BthC2c1mut-sgRNA complex with ds36/36 at 37^°^C.

To prevent the cleavage of the substrate DNA strand by BthC2c1 during the detection process, BthC2c1-D574A/E828A/D952A mutant (mutated at the catalytic sites of the three RuvC domains to deactivate the cleavage activity, referred to as BthC2c1mut) were constructed for affinity detection. The purification method of BthC2c1mut was the same as that of the wild type. The BthC2c1mut-sgRNA complexes were assembled by gel filtration chromatography. The optimal ratio was to mix the BthC2c1 protein with sgRNA at a molar ratio of 1:1.4 SDS-PAGE and denaturing gel electrophoresis results showed that the BthC2c1mut protein and sgRNA appeared together after gel filtration chromatography with a good peak shape (peak A, in Figure **5A**) and the excess RNA was removed (peak B, in Figure **5A**). SDS-PAGE detected the peak fractions, and the fractions of 44, 45, 46, 47 and 48 were collected and prepared for affinity detection (Figures **5C**).

The activity of BthC2c1 was weak at 37°C and significantly enhanced at 42°C. To determine whether this is related to the different affinity of BthC2c1 to substrate at different temperatures, the affinity of BthC2c1mut-sgRNA complex to substrate DNA at 37°C and 42°C was detected by MST. The KD value of the affinity between the BthC2c1-sgRNA complex and ds36/36 at 42^°^C was 7.11 nM. The KD value of the affinity between the BthC2c1-sgRNA and ds36/36 at 37^°^C was 18.69 nM (Figures **5D** and **5E**).

Thus, the affinity of the BthC2c1mut-sgRNA complex to the substrate dsDNA36/36 was significantly enhanced at 42°C than that at 37°C. It is suggested that the binding of BthC2c1mut-sgRNA to dsDNA, especially PAM may be tighter at 42^°^C. Considering that it is different from Cas9 and Cpf1's loose PAM recognition mode, BthC2c1 stringent PAM recognition mode may be part of the reason why BthC2c1 is more active in cleaving substrates at 42°C.

## 4 DISCUSSION

CRISPR-Cas system has become the most useful gene editing technology because of its convenient and fast editing function. Cas9 and Cpf1 have been widely studied and applied in gene editing. C2c1 is a CRISPR-Cas system with smaller molecular weight, lower off-target effects, and higher recognition requirements. It has broad application prospects for gene editing. Through the modification of CRISPR-C2c1, a variety of tools for gene editing have also been obtained. AaC2c1, developed by Teng *et al.,* was effective in introducing targeted indel mutations in mouse embryos and successfully transmitting them to the next generation in germ lines, with no off-target effects observed in either cell lines or mice [20]. Then, four CRISPR-C2c1 systems, AmC2c1, BhC2c1, Bs3C2c1, and LsC2c1, were developed to edit the human genome, and multiple sgRNAs could be used to accurately edit multiple sites in the human genome at the same time [21]. Jonathan *et al.* obtained BhC2c1 v4 by mutating BhC2c1 to promote *in vitro* genome editing of human cell lines and primary human T cells. At the same time, it was also found that BhC2c1 v4 had a higher specificity and lower miss effect than Cas9 [22]. Wu *et al.* used the CRISPR-C2c1 system to edit *Arabidopsis thaliana* and successfully realized multiple genome editing and large fragment gene knockouts [23]. Wang *et al.* successfully edited *Tetraploid cotton* using the CRISPR‐C2c1 system, and no off-target effects were observed after gene sequencing [24].

Our previous studies revealed that the products of BthC2c1 cleave DNA to produce adhesive ends, and the cleavage site is far away from the PAM, which is similar to Cpf1 but different from the adjacent PAM of the Cas9 cleave target DNA site. At the same time, Cas9 NHEJ repair results in a base insertion or deletion, which changes the PAM adjacent sequences and makes Cas9 unable to recognize and cleave target DNA again. However, the DNA cleavage sites of BthC2c1 and Cpf1 are far from the PAM. NHEJ repair does not change the adjacent sequence of the PAM, it can still recognize and cut the target gene, which improves the efficiency of gene editing. In addition, the cleavage sites at which Cas9 and Cpf1 cleave the target DNA are within the heteroduplex of the mediating RNA-target DNA, while the cleavage sites for BthC2c1 are outside the heteroduplex, which has little effect on the heteroduplex. This stable shearing pattern may also apply to achieving multiple cleavages of the same specific site [19].

Based on the previous structural and functional research of the CRISPR‐C2c1 system, BthC2c1 was expressed and purified and the BthC2c1-sgRNA-dsDNA complex was assembled. The effect of temperature on the cleavage ability of the BthC2c1 system was investigated. The interaction of BthC2c1 with sgRNA and dsDNA was detected by molecular sieve. The optimal ratio of assembled complexes is to mix BthC2c1 protein with sgRNA and dsDNA in a molar ratio of 1:1.4:1.5 BthC2c1 cleaves target DNA at a temperature ranging from 37 to 67°C. The cleavage activity of BthC2c1 was extremely weak at 27^°^C and 32^°^C, while it was slightly improved at 37^°^C and significantly enhanced at 42^°^C and 47^°^C. When MST detected the affinity, the affinity of the BthC2c1-sgRNA complex to dsDNA was enhanced at 42°C than that at 37°C. It may be related to the rigid substrate and PAM recognition pattern, which differs from Cas9 and Cpf1. This temperature-related substrate recognition pattern may be part of the reason for the stronger cleavage activity of BthC2c1 at 42°C.

Based on the previous research on the structure and function of the C2c1 system, the BthC2c1 system could be modified, such as truncation and mutation of protein and sgRNA, to improve the editing activity of the system at 37°C and expand its application range. In addition, the higher reaction temperature of BthC2c1system may have certain editing advantages for heat-tolerant plants. Since the cleavage activity of BthC2c1 is related to temperature, the temperature may become a switch for the dynamic regulation of gene editing. This study may provide an experimental basis for optimizing and modifying the C2c1 gene editing system.

## CONCLUSION

BthC2c1 was expressed, purified, and assembled in this study into a complex with sgRNA and dsDNA. BthC2c1 cleaved DNA within the temperature range of 37^°^C to 67^°^C. The affinity of BthC2c1-sgRNA to DNA at 42°C was significantly enhanced than that at 37°C. It may be related to its stringent substrate recognition pattern, which differs from Cas9 and Cpf1. The temperature-dependent affinity changes of substrate binding may be part of the reason for the stronger cleavage activity of BthC2c1 at 42^°^C. This study may provide an experimental basis for optimizing and modifying the C2c1 gene editing system.

## Data Availability

Not applicable.

## LIST OF ABBREVIATIONS

crRNA: CRISPR RNA
tracrRNA: *trans*-activating crRNA
MST: Microscale Thermophoresis
IPTG: Isopropyl β-D-1-thiogalactopyranoside
